# A novel antibacterial and fluorescent coating composed of polydopamine and carbon dots on the surface of orthodontic brackets

**DOI:** 10.1007/s10856-023-06712-8

**Published:** 2023-02-21

**Authors:** Yixi Wang, Chuanyang Ding, Zhangjie Ge, Zhipeng Li, Lixin Chen, Xiaolong Guo, Genxi Dong, Ping Zhou

**Affiliations:** 1 School and Hospital of Stomatology, Lanzhou University, No.222 Tianshui South Road, Chengguan District, Lanzhou, 730000 China; 2 Clinical Research Center of Shaanxi Province for Dental and Maxillofacial Diseases, College of Stomatology, Xi’an Jiaotong University, Xi’an, 710000 China

**Keywords:** Honokiol carbon dots, Polydopamine, Antibacterial coating, Brackets, Reactive oxygen species

## Abstract

Many kinds of antibacterial coatings have been designed to prevent the adherence of bacteria onto the surface of a fixed orthodontic device of brackets. However, the problems such as weak binding force, undetectable, drug resistance, cytotoxicity and short duration needed to be solved. Thus, it has great value in developing novel coating methods with long-term antibacterial and fluorescence properties according to the clinical application of brackets. In this study, we synthesized blue fluorescent carbon dots (HCDs) using the traditional Chinese medicinal honokiol, which could cause irreversible killing effects on both gram-positive and gram-negative bacteria through positive charges on the surface and inducing reactive oxygen species (ROS) production. Based on this, the surface of brackets was serially modified with polydopamine and HCDs, taking advantage of the strong adhesive properties as well as the negative surface charge of polydopamine particles. It is found that this coating exhibits stable antibacterial properties in 14 days with good biocompatibility, which can provide a new solution and strategy to solve the series of hazards caused by bacterial adhesion on the surface of orthodontic brackets.

**Graphical Abstract:**

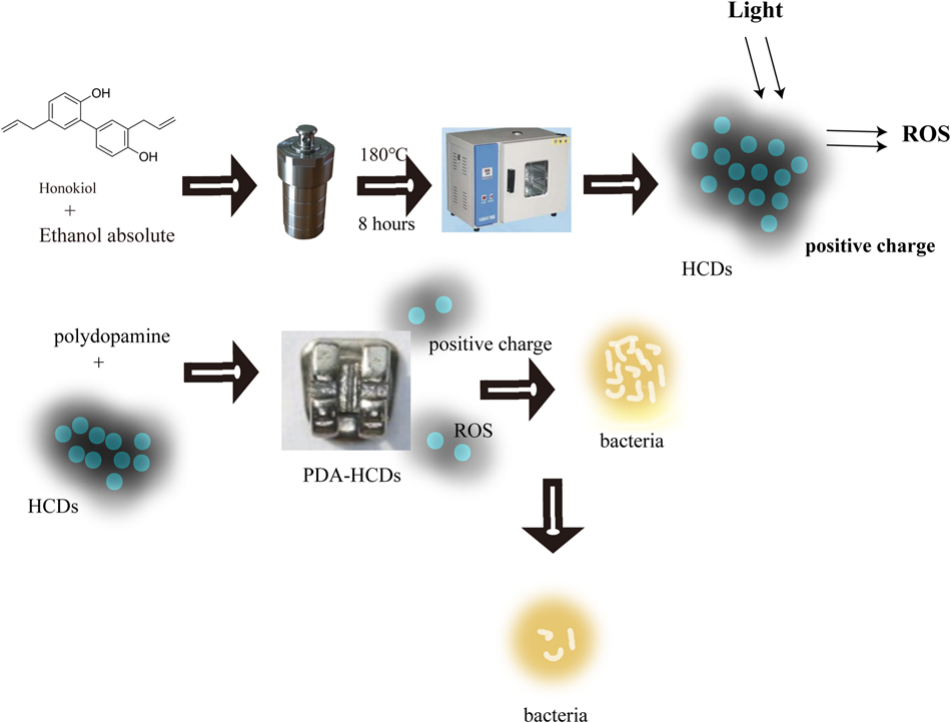

## Introduction

Brackets are widely used in fixed orthodontic treatment. Their complex shape is conductive to food residue retention, which not only brings trouble to the daily oral cleaning of patients but also leads to plaque formation on the tooth surface. These will result in tooth demineralization, caries, gingivitis and periodontitis. In this regard, it is a hot topic to endow antimicrobial abilities to brackets in the field of orthodontics.

Up to now, many kinds of antibacterial reagent loading or stain-resistant coatings have been developed to prepare antibacterial substrates [1–3]. However, most of the stain-resistant coatings lack chemical binding with the substrate surface and exhibit poor mechanical capacities and long-term antibacterial properties. Anti-adhesion polymers mostly require a smooth interface, and such materials have limited antibacterial effects, with no yet elucidated mechanisms [4]. For example, it has been reported that pure silver nanotubes combined with poly (DL-lactic-co-glycolic acid) (PLGA) can inhibit a variety of gram-positive and gram-negative bacteria, but their low adhesion strength and fact degradation result in easy damage [5, 6]. The most commonly reported coatings that release antimicrobial agents include antibiotics, quaternary ammonium salts, antibacterial peptides, and metal ions. Antibiotics exhibit a risk of drug resistance, and the antibacterial ability of quaternary ammonium salts is not good enough. Although silver and copper-based nanomaterials have been widely used in the field of antibacterials, the in vivo biological safety of these metal materials hampers their clinical application [7, 8]. Finally, to our knowledge, it is difficult to release antibacterial peptides from materials with good mechanical properties when decorated onto a solid substrate surface. Notably, most of the current antibacterial coatings do not have fluorescent properties, which are inconvenient for detection and observation. In recent years, scholars have revealed the application prospects of light-mediated antibacterial coatings, whichhave the advantages of fluorescence, a broad antibacterial spectrum, and induction of bacterial resistance and have promise for application in the coating of brackets in comparison to the antibacterial strategies above [9–11].

There is an urgent need to design a coating that strongly binds to the surface of brackets with long-term antibacterial ability and fluorescence. Since 2007, polydopamine (PDA) has been widely used to coat many kinds of solid materials due to its advantages, such as covalent binding, good biocompatibility, hydrophilicity, and ease of decoration or loading of drugs [12]. However, it only exhibits weak photothermal and photodynamic antibacterial ability. To realize clinical application, efforts should be made to graft materials with both good performance in antibacterial and fluorescence. Fortunately, a novel carbon nanomaterial of carbon dots (CDs) that exhibits excellent and unique optical properties, good water solubility, and low toxicity has been reported, and some of them harbor antibacterial properties [13–16]. The antibacterial mechanisms of CDs with positive charges mainly include the generation of reactive oxygen species (ROS), the destruction of bacterial cell walls, the destruction of genomic DNA and so on [17, 18]. It is not easy to induce bacterial resistance, which is very attractive compared to conventional antibacterial drug coatings [19–21].

The hydrothermal method has been the dominant method of synthesizing CDs from various raw materials [22–24]. Different types of CDs can be obtained by changing the precursor carbon source and preparation method for the synthesis of CDs. The synthesized CDs can be modified by atomic doping and group introduction to make them have different structures and properties. Honokiol is a biphenol compound that has antibacterial, antioxidant, anti-inflammatory, antiviral and other properties. It has been reported that Honokiol has broad-spectrum antibacterial activity against both Gram-positive and Gram-negative bacteria, which through its inhibitory effect on the expression of α-hemolysin and Staphylococcus aureus enterotoxins A and B. Compared with traditional antibiotics, antibacterial CDs are less prone to bacterial resistance and have the advantages of low toxicity and high biosafety [25].

In this study, honokiol was applied as a carbon source to prepare positively surface charged CDs using a one-step hydrothermal method, which not only harbor good sustained antibacterial ability against *E. coli* and *S. mutans* through novel physiochemical mechanism, but also could covalently decorate onto the surface of orthodontic brackets with the help of PDA coating. Our fluorescent PDA-HCDs coatings on the bracket surfaces using green raw materials exhibited excellent in vitro safety, and has good mechanical potential to be applied in oral. Our study laid an important foundation for applying CDs based antibacterial coatings to prevent the bacterial adhesion on the surface of orthodontic brackets, and achieve periodic replacement according the fluoresce detection.

## Materials and methods

### Materials

Dopamine hydrochloride, tris[hydroxymethyl]am-inomethane (Tris), Honokiol, Dimethyl sulfoxide (DMSO), 1,3-Diphenylisobenzofuran (DPBF), WST-8 kit, and LIVE/DEAD™ BacLight™ Bacterial Viability L7012 Kit were purchased from Aladdin (Shanghai, China). The Biotech cellulose ester (CE) membrane (MWCO 1000 KD) was obtained from Solarbio (Beijing, China). Difco™ LB broth (Miller), Brian Heart Infusion (BHI) and Difco™ agar (granulated) were purchased from Hopebiol (Qingdao, China).

### Synthesis of HCDs

The synthesis of HCDs from Honokiol was carried out using a hydrothermal method according to a reported procedure with slight modifications [26]. Briefly, 1 mM Honokiol was dissolved in 10 mL of anhydrous ethanol with ultrasound treatment and then transferred into a 25 mL Teflon-based autoclave (Bailan Instrument & Equipment, Shanghai, China) for hydrothermal treatment at 180 °C for 8 h. As the reaction was completed, the stock solution of HCDs was centrifuged at 12000 rpm for 12 min. Afterwards, the HCDs solution was further purified using a 0.22 µm syringe filter followed by dialysis (molecular weight cutoff of the membrane = 1 kDa) for 48 h in Milli-Q water. Finally, the dialyzed solution was freeze-dried at −54 °C under vacuum to obtain HCDs powder.

### Preparation of HCDs-PDA coating on the surface of brackets

Tris·HCl **(**0.0242 g) was dissolved in 20 mL of deionized water, and the pH of the solution was adjusted to 8.5. Then, 0.04 g of dopamine was added to the solution and stirred to obtain polydopamine (PDA) particles. Each of the brackets was placed into each well of a 96-well plate and then coated with these PDA particles using a volume of 150 µL. Unadhered PDA particles were removed by 5 min of ultrasonic cleaning in distilled water. Afterward, 5 mg·mL^−1^ of HCDs solution with different volumes of 50, 100 and 150 µL was reacted onto PDA-coated substrates.

### Characterizations

A drop of the aqueous suspension of nanoparticles was added onto a piece of the carbon-coated copper grid, dried under ambient conditions, and then viewed under a transmission electron microscope (TEM; FEI Tecnai G220 microscope, FEI, Netherland) to examine the nanoparticle morphology. The morphology of the samples was characterized by scanning electron microscopy (SEM; Hitachi S-4800, Japan). The structure was characterized by X-ray diffraction (XRD; MAXima_X 17-XRD6000, China). The surface chemical composition and chemical states of the HCDs were determined by X-ray photoelectron spectroscopy (XPS; Rigaku D/MAX-2400, Thermo Scientific, USA). UV–visible spectrophotometry (RF-5301, Hitachi, Shimadzu, Japan) was used for the characteristic absorption peak. The zeta potentials were determined using a Horiba SZ-100 nanoparticle analyzer (Horiba, Kyoto, Japan) at 25 °C.

### Bacterial culture

Gram-positive Streptococcus mutans (*S. mutans*) and gram-negative Escherichia coli (*E. coli*) were obtained as gifts from the Microbiology Laboratory, School of Stomatology, Lanzhou University. E. coli and S. aureus were cultivated in LB medium containing 10 g·L^−1^ peptone, 5 g·L^−1^ yeast extract, and 10 g·L^−1^ sodium chloride. Then, *S. mutans* were incubated in brain heart infusion medium containing 10 g·L^−1^ protease peptone, 2 g·L^−1^ dextrose, 5 g·L^−1^ sodium chloride, and 2.5 g·L^−1^ disodium phosphate. All samples were kept in an incubator containing 5% CO_2_ for 24 h at 37 °C. The concentration of the bacterial suspension was controlled at 1 × 10^6^ CFU·mL^−1^ by measuring the OD at a wavelength of 600 nm. The OD_600_ value of the bacterial suspension was adjusted to approximately 0.1. The bacterial suspension (OD_600_ = 0.2 ± 0.02) was used directly or diluted to different concentrations for the subsequent tests.

### Antibacterial activity assays for HCDs

To investigate the bacteriostatic characterization of Honokiol and HCDs against two bacterial species, *E. coli* and *S. mutans*, the operation steps were as follows. First, the doubling dilution method was repeated in 96-well plates. Second, 10 µL of bacterial suspension with a concentration of 1 × 10^6^ CFU·mL^−1^ was added to the 96-well plates. The 96-well plates were incubated in 37 °C biochemical incubators for 24 h. Then, a WST-8 kit was added according to the instructions (the ratio of reagent to the bacterial solution was 1:10) for 1 h. Finally, the OD_600_ value of LB broth in the 96-well plate was measured by a Thermo Scientific Microplate Reader.

### The antibacterial ability of PDA-HCDs decorated brackets

In this work, sterilized PDA coated Brackets and PDA-HCDs coated brackets were soaked in artificial saliva for 0, 7 and 14 days, and then the antimicrobial properties of the soaking solution against *E. coli* and *S. mutans* were verified. The operation steps were as follows: 1) PDA-modified Brackets or artificial saliva-soaked PDA-HCDs coated Brackets were placed for 0, 7 and 14 days in a 96-well plate, with pure Brackets as the control; 2) 150 μL of the bacterial solution was added to each well, completely submerged the Brackets, and incubated in a constant temperature incubator at 37 °C for 24 h; 3) the bacterial solution was discarded, each tray was rinsed with 2 mL of saline, the rinse solution of each group of trays was collected in a 96-well plate separately, and saline was used as the background group; 4) the activity of bacteria was detected using a WST-8 kit as mentioned above.

A 12 mm diameter slide was treated with artificial saliva for 2 h to form a biofilm on the surface of the slide, which was placed on a 24-well plate. A total of 300 µL of bacterial suspension (1 × 10^6^ CFU·mL^−1^) and 300 µL of different concentrations of HCDs (0, 12.5, 25, 50, 100, and 500 µg·mL^−1^) were mixed in a 24-well plate. After incubating at 37 °C for 12 h. Aspirate the bacterial solution from the well plate, add the prepared L-7012 reagent to the well to submerge the slide, incubate for 30 min under protection from light, remove the cell crawling tablets, rinse gently with PBS 2 times, blow dry the slide and place it under a fluorescence microscope for observation.

### ROS generation test

The performance of HCDs in ROS generation was quantified by DPBF assay. DPBF is reduced by ROS to form 1,2-dibenzoylbenzene (DBB) with a maximum UV − vis absorption band at a wavelength of 410 nm. Briefly, 1 mL of 20 µg·mL^−1^ DPBF solution was mixed with 1 mL of 2 mg·mL^−1^ HCDs solution (PBS buffer). The mixture was incubated in the dark at room temperature under shaking (150 rpm). Then, at the desired time points (5, 10, 15, 20, 25, 30 min), the absorbance of these mixture solutions was measured by a Hitachi U3900 UV − vis spectrophotometer.

### In vitro biocompatibility of PDA-HCDs coating

The in vitro biocompatibility tests were performed using a cell counting kit 8 (CCK-8) assay on L929 cells. Briefly, impregnation solution for PDA-HCDs coating modified brackets and L929 cells were seeded into 96-well plates (5 × 10^3^ cells/well) for 24 h. After coculture with impregnation solution for PDA-HCDs coating modified brackets for 7 days, 14 days of 10 µL of fresh CCK-8 solution was added for further incubation for 1 h. The absorbance of the solution was determined using a microplate reader at 450 nm.

### Quality determination of PDA-HCDs coatings with different thicknesses

To assess the quality of the abovementioned coatings with different thicknesses, we determined the quality of the coatings using a quartz crystal microbalance (QCM) and chips, as we mentioned before. Similar to brackets, we prepared PDA-HCDs decorated QCM chips using various volumes of HCDs solution. The frequency changes of these QCM chips before and after immersion in artificial saliva for 7 or 14 days were measured. The QCM is based on the piezoelectric influence, where a deposited mass is registered as changes in the frequency of an oscillating QCM crystal. The adsorbed mass can then be calculated by the Sauerbrey equation as we previously described [27].

### Statistical analysis

All the experimental data were statistically analyzed, and the results are expressed as the mean ± standard deviation (SD). Statistical differences were determined using one-way ANOVA followed by a Bonferroni post hoc test for multiple comparisons with SPSS, version 24 (IBM). Data were considered statistically significant when *p* < 0.05 versus the indicated group.

## Results

### Morphology and structure characterization of HCDs

**Fig. 1:**
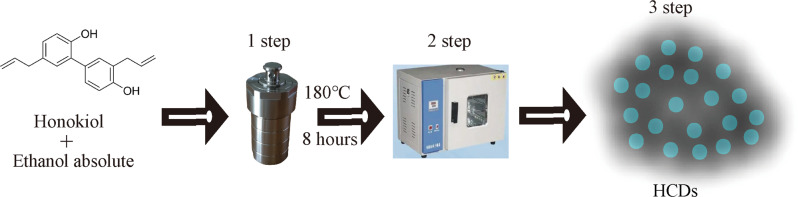
Graphical description for the preparation of HCDs from Honokiol using a hydrothermal method

**Fig. 2:**
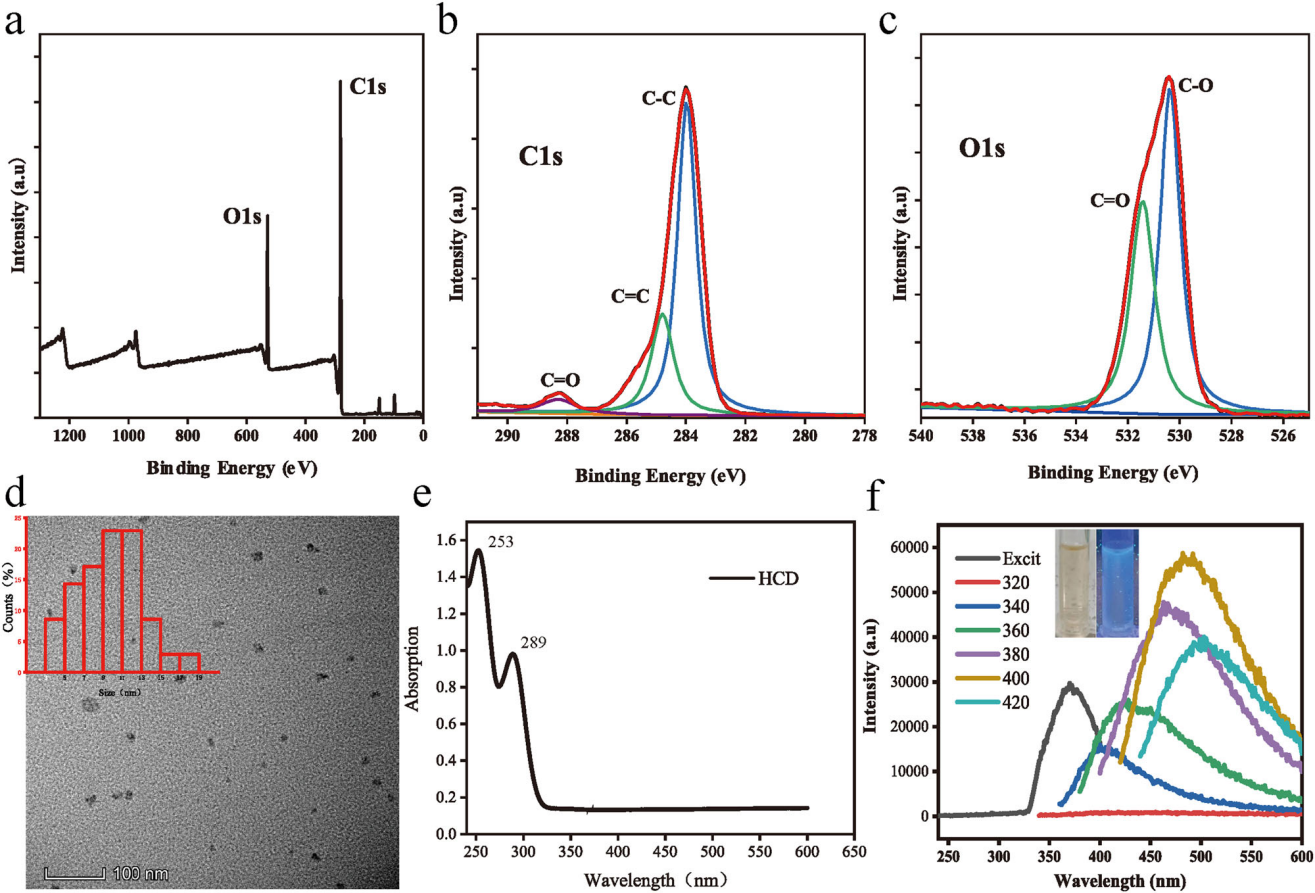
Characterization of HCDs. **a**–**c** The full XPS spectrum of HCDs, and high-resolution XPS spectra of the prepared HCDs (**b**) C1 s, (**c**) O1 s are shown. **d** TEM images of HCDs. **e** UV‒Vis absorption spectrum of HCDs. **f** PL spectrum of obtained HCDs under different excitation wavelengths. The inset images of the HCDs solution under daylight (left) and 365 nm (right) UV irradiation. Scale bar: 100 nm

The preparation process of HCDs from Honokiol is shown in Fig. 1. Honokiol mixed with ethanol was placed into a hydrothermal reactor for 8 h at 180 °C, and HCDs were obtained by centrifugal filtration for the following experiments. XPS elemental analysis was used to analyze the chemical constituents of the HCDs (Fig. 2a). Carbon (C1 s, 63.9%) and oxygen (O1 s, 36.1%) were the dominant components. Then, the high-resolution spectrum of C1 s could be deconvoluted into three curves with binding energies centered at 284.0, 284.8, and 288.3 eV, which correspond to C1 s in the states of C-C, C = C and C = O, respectively (Fig. 2b) [28, 29]. For O1 s, two binding energy peaks at 530.4 and 531.4 (eV) were found in its high-resolution spectrum, indicating that oxygen atoms are present in the forms of C-O- and C = O, respectively (Fig. 2c) [29, 30]. Notably, a certain amount of carbon dioxide is generated. It is well known that these chemicals can decompose to CO_2_/NH_3_ at high temperatures in a closed container. The formed CO_2_/NH_3_ in the autoclave provided an environment with high pressure, which could accelerate the decomposition of organic molecules into small carbon nuclei. Carbon nuclei are composed of sp^2^ carbons and functionalized with different hydrophilic groups, such as such as OH, C = O, NH, C-O etc [1, 31]. Finally, the morphology and hydrodynamic size distribution of the HCDs were detected by TEM (Fig. 2d). The particles were well dispersed and exhibited a nearly spherical shape. The size of the HCDs ranged from 3 nm to 19 nm, with an average size of 11 nm, as measured using ImageJ software (Fig. 2d insert).

### Photoluminescence properties

To investigate the optical properties of our HCDs, UV–Visible absorption and photoluminescence spectrometer analyses were performed (Fig. 2e). The ultraviolet absorption peak of the HCDs appeared between 200 and 300 nm, and the characteristic ultraviolet absorption peaks appeared at 253 nm and 289 nm. HCDs in dimethyl sulfoxide (DMSO) present a yellowish color under daylight but emit blue fluorescence under ultraviolet lamp irradiation (Fig. 2f insert) with not good fluorescence intensity. Figure 2f shows the corresponding emission wavelength of the HCDs with changes in the excitation wavelength. It is found that excitation at an approximately 400 nm wavelength contributed the highest emission at 486 nm wavelength.

### HCDs retained similar antibacterial ability to raw material

**Fig. 3:**
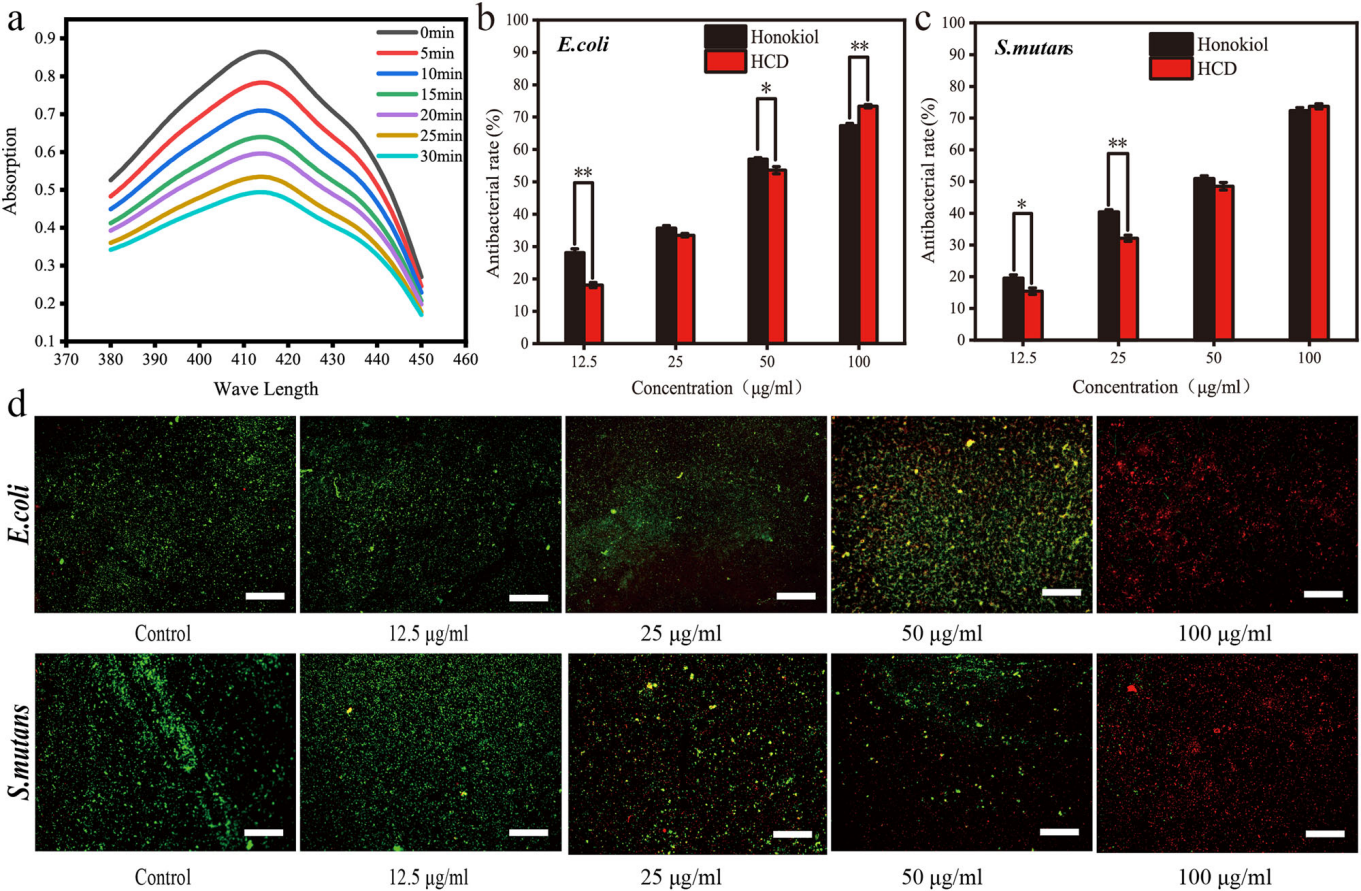
The antibacterial properties of HCDs. **a** The UV absorption spectrum between 350 nm and 500 nm was measured for HCDs by a UV absorption spectrometer under daylight for 0, 5, 10, 15, 20, 25, and 30 min, respectively. **b**, **c** The antibacterial rate of Honokiol and HCDs against *E. coli* (**b**) and *S. mutans* (**c**) as a function of concentration (12.5, 25, 50, 100 μg·mL^-1^) was detected using a WST-8 kit after 24 h of culture. The measured OD_600_ was used to calculate the antibacterial rate using pristine culture as a control. **d** L-7012 reagents were applied for the observed live and dead cells at excitation wavelengths of 488 nm and 594 nm, respectively. *p < 0.05, **p < 0.01. *n* = 3

To investigate the antibacterial potential of our HCDs, analyses of zeta potential and reactive oxygen species (ROS) generation were performed. The zeta potential measured by the average of the HCDs was +27.63 mV, proving that our HCDs surface carries a high positive charge. Moreover, we analyzed the ability of HCDs to produce ROS. We used a probe of 1,3-diphenylisobenzofuran (DPBF) with photostability and fluorescence properties to detect ROS production according to previously reported procedures. 1,3-Diphenylisobenzofuran (DPBF) is a widely used ROS indicator that depends on the absorbance at 410 nm. It bears the merits of a fast response and the absence of ROS generation [32, 33]. When DMF, DMSO and ethanol were used as solvents, the absorption of DPBF did not change significantly under indoor sunlight [34]. Therefore, DPBF can be used to detect ROS production in these solvents. In the presence of ROS, the DPBF probe was activated and reacted with ROS, and eventually DPBF was consumed, thus reducing the absorption value at 410 nm in the UV absorption spectrum. As shown in Fig. 3a, the real-time ultraviolet absorption spectrum of HCDs solution and 1,3-diphenyl isobenzofuran (DPBF) mixture between 350 nm and 500 nm wavelength was detected as a function of natural illumination time. The UV absorption values at 410 nm curve peaks markedly decreased with the augment of natural light in 30 min, confirming the successful production of ROS. These results confirmed the feasibility for subsequent antibacterial applications.

The antibacterial properties of HCDs against gram-positive *E. coli* and gram-negative *S. mutans* were determined after 24 h of incubation using a WST-8 kit, and Honokiol raw material was used as a control (Fig. 3b, c). Using concentrations below 25 μg·mL^−1^, the antibacterial activities of HCDs against both bacteria were slightly weaker than those of Honokiol. However, when the concentration was increased to 50 μg·mL^−1^, the HCDs exhibited antibacterial ability similar to that of honokiol and even showed significantly better performance against *E. coli* at 100 μg·mL^−1^ (*p* = 0.00071). Notably, for both bacteria, more than 50% of bacteria were killed at 50 μg·mL^−1^, and the rate values increased to 70% at 100 μg·mL^−1^. Moreover, live/dead cell staining was performed to visualize the biological activity of HCDs against these bacteria (Fig. 3d). Living and dead bacteria, respectively, present red and green fluorescence. Consistent with the WST-8 assay, the number of live bacteria, especially *S. mutans*, was apparently reduced with increasing concentrations of HCDs. Finally, many dead bacteria with quite a few green signals were observed in the 100 μg·mL^−1^ group. These results demonstrated the good antibacterial activity of HCDs against both gram-positive and gram-negative bacteria through previously mentioned mechanisms that do not induce bacterial drug resistance.

### Fabrication of polydopamine and HCDs coatings on the surface of brackets

**Fig. 4:**
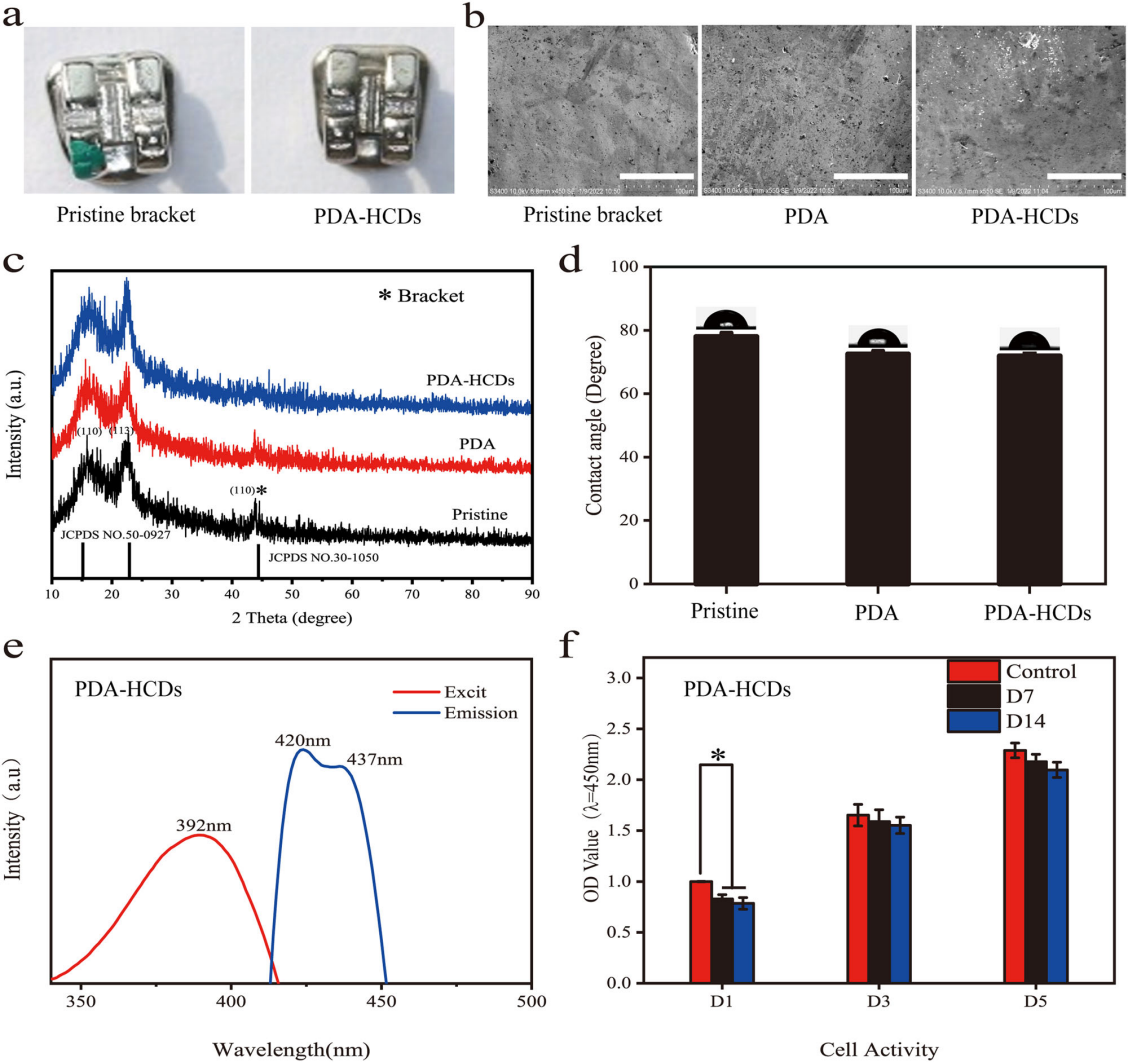
Characterization of PDA-HCDs coatings on the substrate surface. **a** Photos of the pristine bracket (left) and the bracket that was modified with the PDA-HCDs coating (right). **b**, **c** Analyses of SEM (**b**) and XRD (**c**) for a pristine bracket, PDA-bracket, PDA-HCDs bracket. **d** Water contact angle measurements for these substrates. **e** Analyses of excitation and emission spectra for the PDA-HCDs decorated bracket. **f** PDA-HCDs decorated substrates were soaked in cell culture medium for 7 days and 14 days, and L929 cells were co-cultured with soaking solutions, respectively. The viabilities of cells after incubations for 1, 3, and 5 days were measured using a CCK-8 reagent at a 450 nm wavelength. *p < 0.05, **p < 0.01. *n* = 3

The surface of the brackets was subsequently coated by electronegative PDA particles and HCDs through electrostatic adsorption (Fig. 4a). Meanwhile, various volumes (50 µL, 100 µL and 150 µL) of the HCDs solution were uniformly pipetted onto the PDA coating surface and allowed to dry. The surface morphology of the pristine bracket surface and PDA-HCDs (100 µL)-coated substrates was evaluated by SEM (Fig. 4b), and more particles were found on the surface of the brackets after surface modifications (Fig. 4b). Then, XRD analyses showed a characteristic peak located at 43.9^o^, which corresponds to the brackets. The characteristic peak of brackets was weakened after the PDA coating and missed with further decoration of HCDs, suggesting that the surface was almost fully covered by the PDA-HCDs coatings. Because the bracket used in our experiment is stainless steel, the main component is iron, and its corresponding JCPDS number is NO.30-1050. The peaks at 15.182° and 22.89° are characteristic peaks of carbon in stainless steel, and its corresponding JCPDS number is NO.50-9027. As shown in Fig. 4d, the water contact angles of the bracket surface were reduced after the decoration of PDA and remained nearly the same for the final PDA-HCDs coated substrates. Similar to the mussel adhesion mechanism, PDA can adhere to the surface of most substances and has been used in biomedicine to increase the hydrophilicity of material surfaces [35, 36]. The increased hydrophilicity can promote bacterial adhesion and facilitate the bactericidal effect of HCDs, thus further improving the antibacterial performance of HCDs [37, 38].

Finally, it was found that the excitation spectra peaked at 392 nm for the PDA-HCDs coating and resulted in broad emissions with peak values ranging from 420 nm to 437 nm. These results indicated that PDA-HCDs coating endows the surface with fluorescent properties. In addition, PDA-HCDs coated brackets were incubated in culture medium for 7 and 14 days, and the extract liquids were collected for the culture of L929 cells to study biocompatibility. As shown in Fig. 4f, no obvious cytotoxicity was detected during the 5 days of culture, proving that the PDA-HCDs coating had good biosafety when placed onto the tooth surfaces.

### The HCDs-PDA coating showed long-term antibacterial activity on the bracket surface

**Fig. 5:**
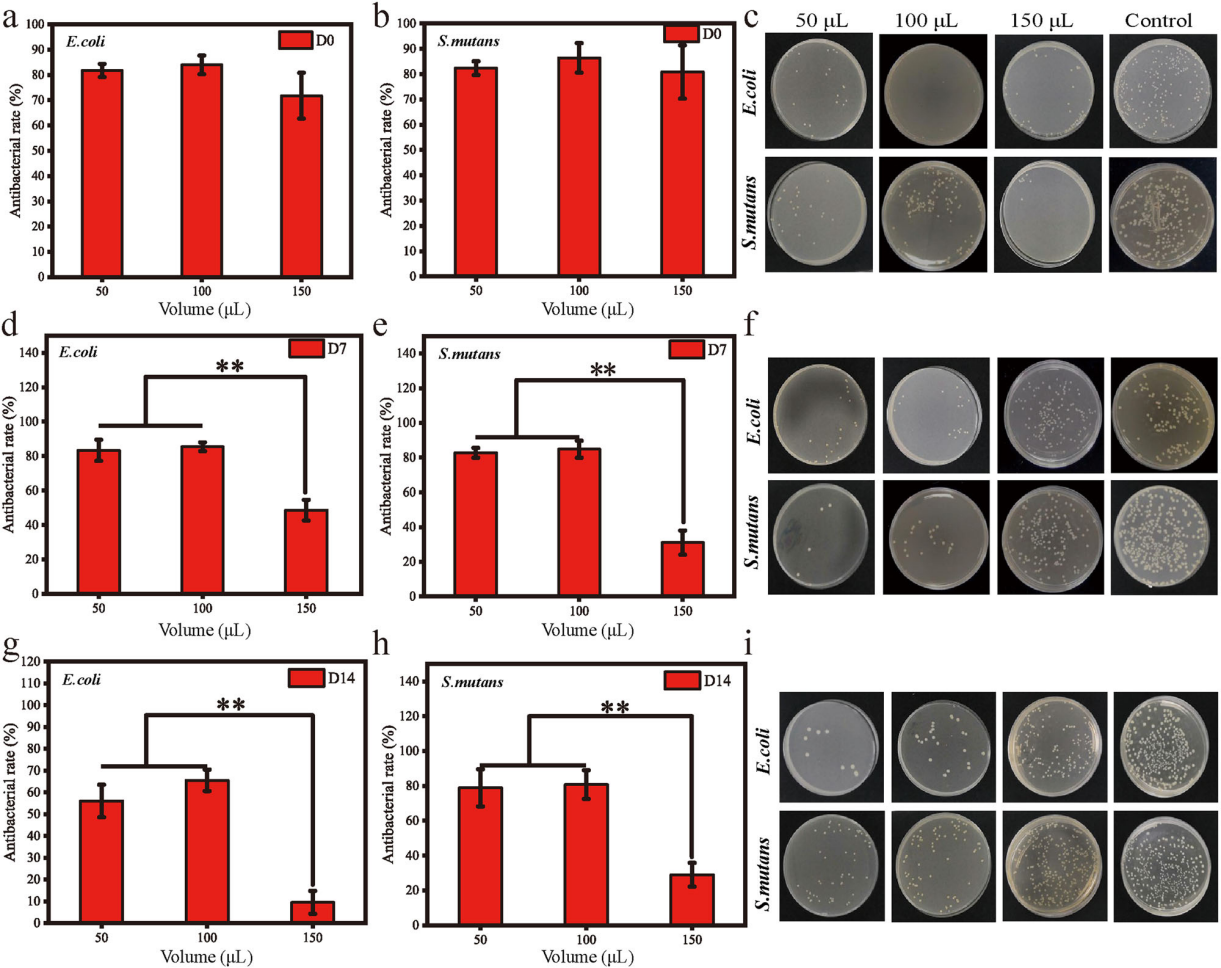
Long-term antibacterial performance of PDA-HCDs coating. **a**, **b** The antibacterial rates of PDA-HCDs coatings with different volumes of HCDs solutions (50 µL, 100 µL and 150 µL) against *E. coli* (**a**) and *S. mutans* (**b**). Pristine brackets were used as controls. **c** The corresponding bacterial plate counting results for these samples. **d**–**i** Similar antibacterial experiments were performed after soaking in artificial saliva for 7 days and 14 days for those samples. **p < 0.01. *n* = 3

After immersion in artificial saliva for varying numbers of days (0, 7, and 140), a WST-8 kit was also used to evaluate the antibacterial properties of brackets that were modified with HCDs-PDA coatings (Fig. 5). First, consistent with reported results, we confirmed that the PDA coating showed nearly no effect on the culture of bacteria on the substrate surfaces (Fig. S1). Then, it is shown that all three groups with different thicknesses have high antibacterial rates against *E. coli* and *S. mutans* before immersion. In addition, the antibacterial rate of the coating group formed by 150 μL PDA-HCDs solution was slightly lower than that of the other two groups (*p* > 0.05). After 7 days of soaking, similar antibacterial rates, more than 80%, of *E. coli* and *S. mutans* were detected in the 50 μL and 100 μL groups. Both were remarkably higher than those in the 150 μL group (*p* < 0.05). Similar results with an increased difference were found with the prolongation of soaking days to 14 days. Fortunately, at this time point, the antibacterial rates of the 50 μL and 100 μL groups against both bacteria could still reach more than 50%. Finally, consistent plate count results were observed for these groups at each desired time point (Fig. 5b, d, f). The number of colonies of *E. coli* and *S. mutans* in the groups with PDA-HCDs coatings was much smaller than that in the control group. After soaking in artificial saliva for 7 days and 14 days, the coatings formed by 50 μL and 100 μL HCDs still exhibited good antibacterial properties, but the antibacterial properties of the coatings formed by 150 μL HCDs were much weaker than those of the above two groups.

QCM equipment and QCM chips were applied to evaluate the stability of PDA-HCDs coating on the substrate surfaces (Fig. S2). Similar to the bracket substrates, PDA-HCDs coating was prepared on the QCM chips. Then, the mass changes of these QCM chips after 7 days and 14 days of incubation in artificial saliva were investigated. As a matter of course, the mass of PDA-HCDs coating was positively correlated with the volume of the mixture solution. Surprisingly, 50 µL and 100 µL of the HCDs solution contributed similar mass changes of the QCM chips on day 7, but much higher than that coated with HCDs with a volume of 150 µL. The same results were found after 14 days of incubation. Notably, the mass changes of the QCM chips remained almost constant for the 50 µL and 100 µL groups during the second 7 days of incubation. These results suggested that the HCDs coating with volumes of 50 µL and 100 µL were more stable than the 150 µL group after 14 days when decorated on the PDA coating surfaces. These results could explain why the 150 µL group showed weaker antibacterial abilities than the other two groups on both days 7 and 14, as described previously. The stability of the coating will decrease when the thickness exceeds a certain value for HCDs, and a suitable thickness with a volume of 50–100 µL maintains good antibacterial activity for a long time.

## Discussion

At present, the problem of tooth damage caused by bacterial growth on the surface of the bracket is still a huge challenge for dentists. As described above, CDs have unique advantages in visualizing and killing bacteria. In 2018, an antibacterial coating of ZnO/CDs composite was applied to modify orthodontic brackets [39]. However, the lack of strong binding with the substrate surfaces, as well as the weak antibacterial ability, impede its clinical application. In addition, the effect of the oral saliva environment on those performances has not been deeply studied. In this study, we synthesized fluorescent CDs (HCDs) from the traditional Chinese medicinal honokiol and then combined them with negatively surface-charged PDA particles to generate a stable coating on the bracket surfaces (Fig. 1).

The prepared HCDs showed not good blue fluorescence under ultraviolet light (Fig. 2f insert), which is supposedly due to the absence of heteroatom doping during the synthesis process. Fortunately, our results confirmed that HCDs exhibited good antibacterial abilities against both gram-positive and gram-negative bacteria (Fig. 3). To our knowledge, the antibacterial properties of CDs depend on size, shape, functional group, surface charge, and the nature of microorganisms [18]. The surface charge of CDs can be divided into positive, negative or electrically neutral. It has been reported that positively charged CDs harbor broad-spectrum antibacterial activity through strong electrostatic interactions with the negatively charged cell membrane surface, which further disrupts their integrity and leads to the leakage of cytoplasmic organelles [40].

The bisphenol structure of the raw material was destroyed after the hydrothermal treatment. However, research shows the precursors of CDs appreciably impact the antibacterial activity and bacteriostatic species. CDs could preserve some carbon source characteristics during the carbonization process [41]. Fortunately, our HCDs possess a great positive charge with a zeta potential of +27.63 mV. It is reported that the kind and ratio of raw materials as well as the change in carbon point synthesis conditions can control the surface charge of CDs, which can be shown as positive charge, negative charge and neutral charge [42, 43]. Therefore, we speculate that the positive charge on the surface of HCDs may be due to the formation of specific carbon nuclei and the action of surface groups under specific high-temperature carbonization conditions. These results indicated that HCDs possess great promise for antibacterial applications. More importantly, CDs can generate reactive oxygen species (ROS) to produce oxidative stress to kill bacteria. Bing et al. reported that the positive CDs induced ROS generation higher than that of negative CDs for the disruption of the bacterial cell membrane, but workless for the neutral ones [42]. Based on this, our HCDs hold the potential to induce the production of ROS under natural light, following the processes of singlet oxygen penetrating the bacterial cell membrane, protein inactivation, nucleic acid destruction, and lipid peroxidation, thereby producing irreversible effects on bacteria [44–46]. As shown in Fig. 3a, HCDs successfully induced rapid ROS production and oxidative stress. disrupting the balance of the cell membrane and leading to the leakage of cytoplasmic material [47]. These results demonstrated that our electropositive HCDs could not only directly damage the bacterial cell membrane but also generate ROS to kill the bacteria, exhibiting great promise for application as a fluorescent antibacterial agent.

Afterwards, CDs were combined with PDA particles to generate the HCDs-PDA composite, followed by the formation of coatings on the surfaces of the bracket with changing solution volumes (50 µL, 100 µL, and 150 µL) (Fig. 4). We further investigated the long-term antibacterial activity of these substrates after soaking in artificial saliva for different numbers of days (0, 7 and 14). The results showed that the HCDs-PDA coatings using volumes of 50 and 100 µL exhibited stable and good antibacterial activity after soaking for more than 7 days, which was significantly better than both the pristine control and 150 µL groups (Fig. 5, *p* < 0.01).

These results demonstrated that our HCDs-PDA coatings harbor excellent antibacterial abilities over a long period, and the binding force between the HCDs-PDA particles and the bracket surface seems stronger than the particles themselves.The binding of PDA on the surface of brackets is mainly due to the coordination between the o-diphenol hydroxyl structure of PDA and metal atoms on the surface of the brackets [48]. In addition, the existence of hydrogen bonds also increases the binding force. The abundant positive charge on the surface of the HCDs makes it bind to the surface of the PDA coating by electrostatic interactions and physical adsorption. Consistently, the quantitative results of evaluating the stability of PDA-HCDs coatings on the substrate surface using QCM devices and QCM chips also confirm our conjecture (Fig. S2). In addition, we explored the antibacterial properties of PDA decorated brackets PDA coatings alone, and only slight effects on the growth of *E. coli* and *S. mutans* were measured (Fig. S1) [49]. This suggested that the antibacterial properties of PDA-HCDs coating mainly come from HCDs on bacteria, and PDA mainly adhered HCDs stably to the surface of brackets through its excellent adhesion properties, thereby forming brackets with excellent and stable antibacterial properties. Together, these results indicate that 50–100 μL PDA-HCDs particles can form a stable coating on the bracket surface, maintaining long-term antibacterial activities.

Our antibacterial PDA-HCDs coating makes the surfaces of brackets fluorescent, which can be detected by the equipment. Moreover, its good biosafety was proven by an in vitro cell experiment (Fig. 4f), suggesting that the modified bracket is not harmful when placed onto the teeth in the human mouth. Notably, it is difficult to distinguish with the naked eye, which should be because there are no atoms such as N and S doped in the process of synthesizing HCDs [50]. This gives us the hope of further developing visual antibacterial coatings with naked-eye fluorescence. We will make further effort to prepare CDs based coatings on the surface of brackets with bright fluorescence by improving the fluorescence performance of antibacterial CDs.

In summary, HCDs harbor a superior antibacterial ability in killing bacteria through positive charges effect and the generation of ROS. Simultaneously, these mechanisms make HCDs have a low probability of inducing the development of bacterial drug resistance [51, 52]. Then, the combination with PDA particles realizes the grafting of CDs onto substrate surfaces with strong binding performance and good antibacterial ability. The results of the long-term antimicrobial activity of the PDA-HCDs coating on the bracket surface seem to be more convincing. The fluorescence properties of the PDA-HCDs coating on the bracket surface can be captured with instrumentation, which guides us to further develop fluorescence visualization of the antimicrobial coating.

## Conclusions

In the present study, we successfully prepared antibacterial HCDs with blue fluorescence from honokiol using a hydrothermal method. HCDs showed positively surface charged with the ability of ROS production. Then, an antibacterial coating of PDA-HCDs with good biocompatibility was developed on the bracket surface through electrostatic adsorption, which success to exhibit long-term antibacterial activity against *E. coli* and *S. mutans*. These result shows the great potential of CDs coatings as antibiotic-free antibacterial agents and monitors functionalized nanoplatform candidates for the decoration of brackets in clinical practice.

## Supplementary Information

Supporting information
