# Supplementary material for: A novel antibacterial and fluorescent coating composed of polydopamine and carbon dots on the surface of orthodontic brackets
Source: J Mater Sci Mater Med. 2023 Feb 21;34(2):10. doi: 10.1007/s10856-023-06712-8 (PMC9943946; doi:10.1007/s10856-023-06712-8)
Supplement: Supplementary file 1 — Supporting information [file 10856_2023_6712_MOESM1_ESM.docx]

**Supporting information for**

**A novel antibacterial and fluorescent coating composed of polydopamine and carbon dots on the surface of orthodontic brackets**

Yixi Wang^1,a^, Chuanyang Ding^1,a^, Zhangjie Ge^1,a^, Zhipeng Li ^a^, Lixin Chen ^a^, Xiaolong Guo ^a^, Genxi Dong ^a,^*, Ping Zhou ^a,b,^*

^a^ School and Hospital of Stomatology, Lanzhou University, No.222 Tianshui South Road, Chengguan District, Lanzhou 730000, China

^b^ Clinical Research Center of Shaanxi Province for Dental and Maxillofacial Diseases, College of Stomatology, Xi’an Jiaotong University, Xi’an 710000, China

**Corresponding author**

*Address correspondence to Prof. Ping Zhou (e-mail: zhoup@lzu.edu.cn) or to Prof. Genxi Dong (e-mail: gxdong@lzu.edu.cn).

1 These authors contributed equally to this study.

**Supporting figures**


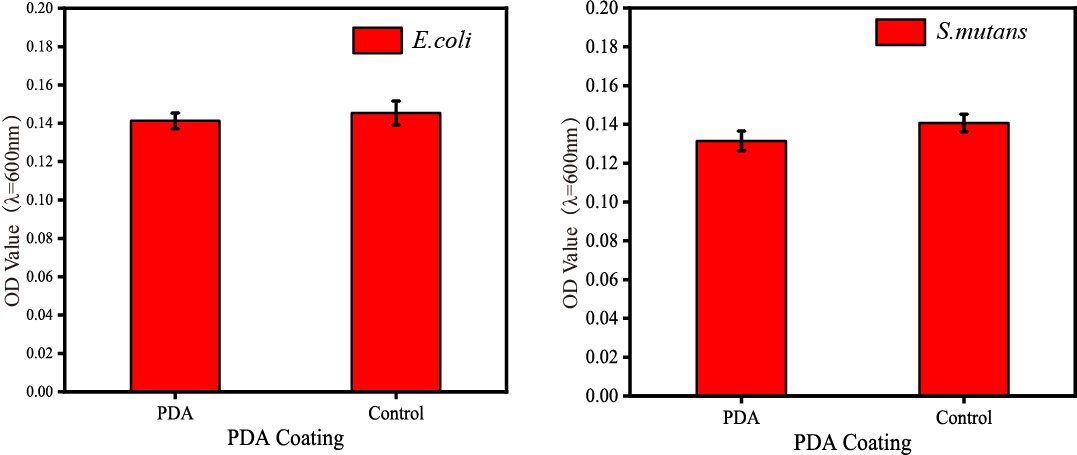


**Figure S1** Antibacterial properties of PDA modified brackets. PDA coated brackets and pure brackets were incubated with *E. coli* and *S. mutans* in a constant temperature incubator for 24 hours. and then added to WST-8 kit. The measured OD_600_ was used to evaluate antibacterial effect. n=3.


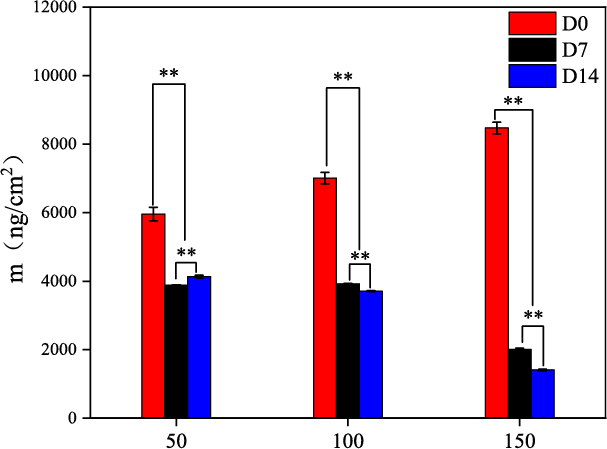


**Figure S2** Mass changes of PDA-HCDs coating. The mass change of QCM chips decorated by PDA-HCDs coatings with varying volumes of HCDs solutions (50 µL, 100 µL and 150 µL) as a function of soaking times. The measurements were performed at both day 0, 7 and 14 days. ** indicates p < 0.01, n=3.
